# Tofacitinib inhibits granulocyte–macrophage colony-stimulating factor-induced NLRP3 inflammasome activation in human neutrophils

**DOI:** 10.1186/s13075-018-1685-x

**Published:** 2018-08-29

**Authors:** Makiko Yashiro Furuya, Tomoyuki Asano, Yuya Sumichika, Shuzo Sato, Hiroko Kobayashi, Hiroshi Watanabe, Eiji Suzuki, Hideko Kozuru, Hiroshi Yatsuhashi, Tomohiro Koga, Hiromasa Ohira, Hideharu Sekine, Atsushi Kawakami, Kiyoshi Migita

**Affiliations:** 1 Department of Rheumatology, Fukushima Medical University School of Medicine, 1 Hikarigaoka, Fukushima, Fukushima 960-1295 Japan; 2 Department of Rheumatology, Ohta Nishinouchi General Hospital Foundation, 2-5-20 Nishinouchi, Koriyama, Fukushima, 963-8558 Japan; 3 Clinical Research Center, NHO Nagasaki Medical Center, Kubara 2-1001-1 Omura, Nagasaki, 856-8562 Japan; 4 Department of Immunology and Rheumatology, Unit of Translational Medicine, Graduate School of Biomedical Sciences, Nagasaki University, Sakamoto1-7-1, Nagasaki, 852-8501 Japan; 5 Department of Gastroenterology, Fukushima Medical University School of Medicine, 1 Hikarigaoka, Fukushima, Fukushima 960-1295 Japan; 6 Department of Immunology, Fukushima Medical University School of Medicine, 1 Hikarigaoka, Fukushima, Fukushima 960-1295 Japan

**Keywords:** Granulocyte–macrophage colony-stimulating factor, Inflammasome, Interleukin-1 beta, Janus kinase, Neutrophils, NLR family pyrin domain-containing 3, Tofacitinib

## Abstract

**Background:**

Granulocyte–macrophage colony-stimulating factor (GM-CSF) has emerged as a crucial cytokine that activates myeloid cells to initiate tissue inflammation. However, the molecular actions of GM-CSF against innate immunity are still poorly characterized. Here, we investigated the *in vitro* effects of GM-CSF on the activation of human myeloid lineages, neutrophils, and the underlying intracellular signaling mechanism, including inflammasome activation.

**Methods:**

Human neutrophils were stimulated with GM-CSF in the presence or absence of tofacitinib. The cellular supernatants were analyzed for interleukin-1 beta (IL-1β) and caspase-1 by enzyme-linked immunosorbent assay (ELISA) methods. Pro-IL-1β mRNA expressions in human neutrophils were analyzed by real-time polymerase chain reaction. Protein phosphorylation of neutrophils was assessed by Western blot using phospho-specific antibodies.

**Results:**

Stimulation with GM-CSF alone, but not tumor necrosis factor-alpha, was shown to increase the release of IL-1β and cleaved caspase-1 (p20) from human neutrophils. Tofacitinib, which inhibits GM-CSF–induced Janus kinase 2 (Jak2)-mediated signal transduction, completely abrogated GM-CSF–induced IL-1β and caspase-1 (p20) secretion from neutrophils. GM-CSF stimulation also induced pro-IL-1β mRNA expression in neutrophils and induced NLR family pyrin domain-containing 3 (NLRP3) protein expression. Although tofacitinib pretreatment marginally inhibited GM-CSF–induced pro-IL-1β mRNA expression, tofacitinib completely abrogated NLRP3 protein expression in neutrophils.

**Conclusions:**

These results indicate that GM-CSF signaling induces NLRP3 expression and subsequent IL-1β production by affecting neutrophils, which may cause the activation of innate immunity. Therefore, GM-CSF is a key regulator of the NLRP3 inflammasome and IL-1β production by activating innate immune cells. This process can be blocked by tofacitinib, which interferes with JAK/STAT signaling pathways.

**Electronic supplementary material:**

The online version of this article (10.1186/s13075-018-1685-x) contains supplementary material, which is available to authorized users.

## Background

Granulocyte–macrophage colony-stimulating factor (GM-CSF) is a hematopoietic growth factor that stimulates the proliferation of granulocytes and macrophages from bone marrow precursor cells [1]. Recent studies suggest that GM-CSF has many pro-inflammatory functions and plays an important role in the development of autoimmune and inflammatory diseases [2]. For example, GM-CSF plays a central role in the pathogenesis of rheumatoid arthritis (RA) by activating the differentiation and survival of macrophages and neutrophils in the rheumatoid synovium [3]. Moreover, a case report showed that the administration of GM-CSF exacerbated RA [4]. Conversely, therapies targeting GM-CSF have been demonstrated to be effective against patients with active RA [5]. These findings suggest that GM-CSF can prime monocyte/macrophage activation and inflammatory cytokine production, leading to a pro-inflammatory network loop that is maintained in rheumatoid synovitis [6].

GM-CSF has also been implicated in the progression of arthritis in mice expressing a transgene encoding human interleukin-1 alpha (IL-1α) [7], indicating a possible link between IL-1 and GM-CSF in inflammatory arthritis. IL-1β is a key cytokine involved in the regulation of immune responses as well as several inflammatory disorders [8]. The secretion of IL-1β, in contrast to that of other inflammatory cytokines, is a tightly controlled two-step process involving the induction of pro-IL-1β and its processing into mature IL-1β by caspase-1, in which NLR family pyrin domain-containing 3 (NLRP3) inflammasome activation plays a critical role [9]. Recent investigations suggest that GM-CSF can act as an enhancer of inflammasome-dependent IL-1β secretion in response to stimuli such as monosodium urate [10]. The GM-CSF receptor is expressed on myeloid cells, including neutrophils. It is a heterodimer of an α-subunit that binds a common β-subunit (c β) [11]. This c β subunit constitutively associates with Janus kinase 2 (JAK2) and undergoes tyrosine phosphorylation prior to the initiation of signaling [12]. Signal transducer and activation of transcription (STAT) is recruited into the cytoplasmic domain of cytokine receptors and is phosphorylated by receptor-associated JAK family kinases [13, 14].

The function of the JAK/STAT signaling pathway in neutrophils and its relationship with IL-1β production are poorly understood. Therefore, this study examined the role of GM-CSF in the cytokine network by determining its effect against neutrophils. We also determined whether an alternation of signal transduction by a JAK inhibitor could regulate the secretion of IL-1β. We report that GM-CSF directly induces IL-1β secretion from neutrophils by activating the NLRP3 inflammasome.

## Methods

### Reagents

Recombinant human GM-CSF and tumor necrosis factor-alpha (TNF-α) were purchased from PeproTech (Rocky Hill, NJ, USA). Anti-β-actin antibodies were purchased from Santa Cruz Biotechnology Inc. (Dallas, TX, USA). Anti-NLRP3 antibody was purchased from Merck Millipore (Billerica, MA, USA). Human IL-1β and caspase-1 (p20) enzyme-linked immunosorbent assay (ELISA) kits were purchased from R&D Systems (Minneapolis, MN, USA). Human IL-18 ELISA kits were purchased from MBL (Nagoya, Japan). Phospho-specific antibodies against JAK-1 (Tyr1022/1023), JAK-2 (Tyr1007/1008), STAT-5 (Tyr701), and STAT-3 (Tyr705) were purchased from Cell Signaling Technology (Beverly, MA, USA). Phospho-specific antibody against JAK3 (Tyr980) was purchased from Santa Cruz Biotechnology (Santa Cruz, CA, USA). Anti-phospho-1κB-α (Ser32), anti-phospho-NF-κB p65 (Ser536), anti-IκBα, and anti-NF-κB antibodies were purchased from Cell Signaling Technology (Danvers, MA, USA). Anti-phospho-specific Asc (Tyr-144) antibody was purchased from ECM Biosciences (Versailles, KY, USA). Anti-caspase-1 antibody (14F468) was purchased from Novus Biologics (Littleton, CO, USA). Tofacitinib was purchased from Sigma-Aldrich (Tokyo, Japan).

### Neutrophil isolation

Venous peripheral blood was collected from healthy volunteers. Written informed consent for blood donation was obtained from each individual. The blood was layered on a Polymorphprep TM (Axis-Shield, Oslo, Norway) cushion and cells were isolated in accordance with the protocol of the manufacturer. Briefly, neutrophils were isolated on the basis of density, washed once in 0.5 N RPMI-1640 to restore osmolality, and then washed once more in RPMI-1640. Using this procedure, we obtained higher purity of CD13^+^ neutrophils. The cells were subsequently diluted in complete medium consisting of RPMI-1640.

### ELISA and Western blot analysis

Neutrophils (2 × 10^6^/mL) were seeded in 24-well plates containing RPMI1640 supplemented with 10% heat-inactivated fetal bovine serum and stimulated with TNF-α or GM-CSF. Cell-free supernatants were collected by centrifugation at 400*g* for 5 min and assayed for IL-1β or caspase-1 (p20) using ELISA kits. Caspase-1 p20 detection was carried out by using a commercially available ELISA kit (Quntikine human caspase-1 p20, R&D Systems) in which monoclonal antibody specific to the p20 subunit of caspase-1 was pre-coated onto a microplate as captured antibody and bounded caspase-1 are detected by another p20-specific polyclonal antibody.

### Reverse transcription–polymerase chain reaction

Total RNA was extracted from neutrophils by using the RNeasy total RNA isolation protocol (Qiagen, Crawley, UK) in accordance with the protocol of the manufacturer. First-strand cDNA was synthesized from 1 μg of total cellular RNA by using an RNA polymerase chain reaction kit (Takara Bio Inc., Otsu, Japan) with random primers. Thereafter, cDNA was amplified by using specific primers respectively. The amplification of the IL-1β transcripts was also accomplished on a Light Cycler (Roche Diagnostics, Mannheim, Germany) by using specific primers. The housekeeping gene fragment of glyceraldehydes-3-phosphates dehydrogenase (GAPDH) was used for verification of equal loading.

#### Cell lysis and Western blotting

Freshly isolated neutrophils were stimulated with GM-CSF (50 ng/mL) for the times indicated in the figure legends, and the cells were washed by ice-cold phosphate-buffered saline and lysed with RIPA Buffer (Sigma-Aldrich) supplemented with 1.0 mM sodium orthovanadate, 10 μg/mL aprotinin, and 10 μg/mL leupeptin for 20 min at 4 °C. After 5 min on ice, the cell lysates were centrifuged at 10,000*g* for 10 min at 4 °C. After centrifugation, cellular lysates (30 μg) were also subjected to 12% SDS-PAGE followed by Western blot with antibodies against human NLRP3 or β-actin with an ECL Western blotting kit (Amersham, Little Chalfont, UK). Only in the signal transduction analysis, cells were pretreated with tofacitinib for 30 min and then stimulated with GM-CSF. Western blot analysis using phospho-specific anti-JAK and STAT antibodies was performed with an ECL Western blotting kit (Amersham).

### Statistical analysis

Differences between groups were examined for statistical significance by using the Student *t* test. *P* values of less than 0.05 were considered statistically significant.

## Results

### GM-CSF induces IL-1β secretion from human neutrophils

**Fig. 1:**
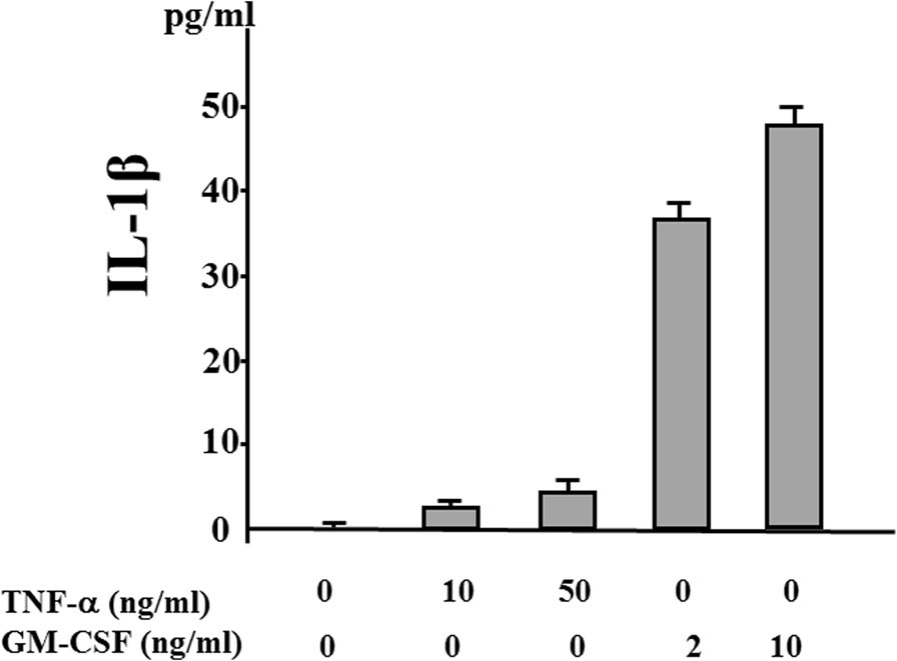
Granulocyte–macrophage colony-stimulating factor (GM-CSF) induces interleukin-1 beta (IL-1β) synthesis from neutrophils. Neutrophils (2 × 10^6^/mL) were incubated with the indicated concentrations of tumor necrosis factor-alpha (TNF-α) or GM-CSF for 24 h, and supernatants were analyzed for IL-1β production by enzyme-linked immunosorbent assay. Values represent the mean ± standard deviation of two independent experiments

**Fig. 2:**
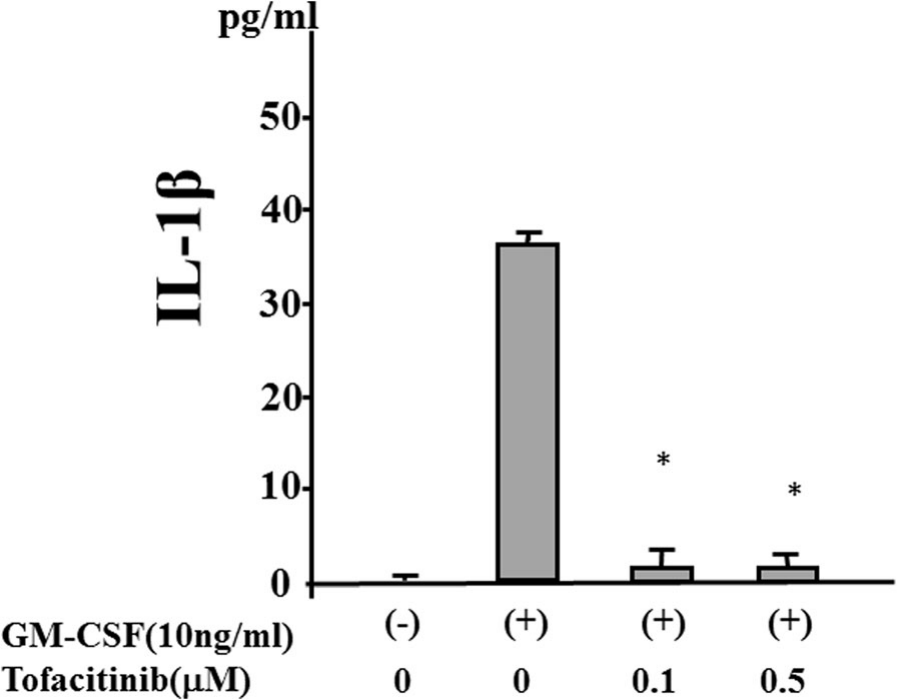
Tofacitinib inhibits the interleukin-1 beta (IL-1β) synthesis from granulocyte–macrophage colony-stimulating factor (GM-CSF)–stimulated neutrophils. Neutrophils (2 × 10^6^/mL) were stimulated with GM-CSF (10 ng/mL) in the presence or absence of the indicated concentrations of tofacitinib for 24 h, and supernatants were analyzed for IL-1β production by enzyme-linked immunosorbent assay. Values represent the mean ± standard deviation of two independent experiments. *P <0.01 compared with GM-CSF–stimulated neutrophils

**Fig. 3:**
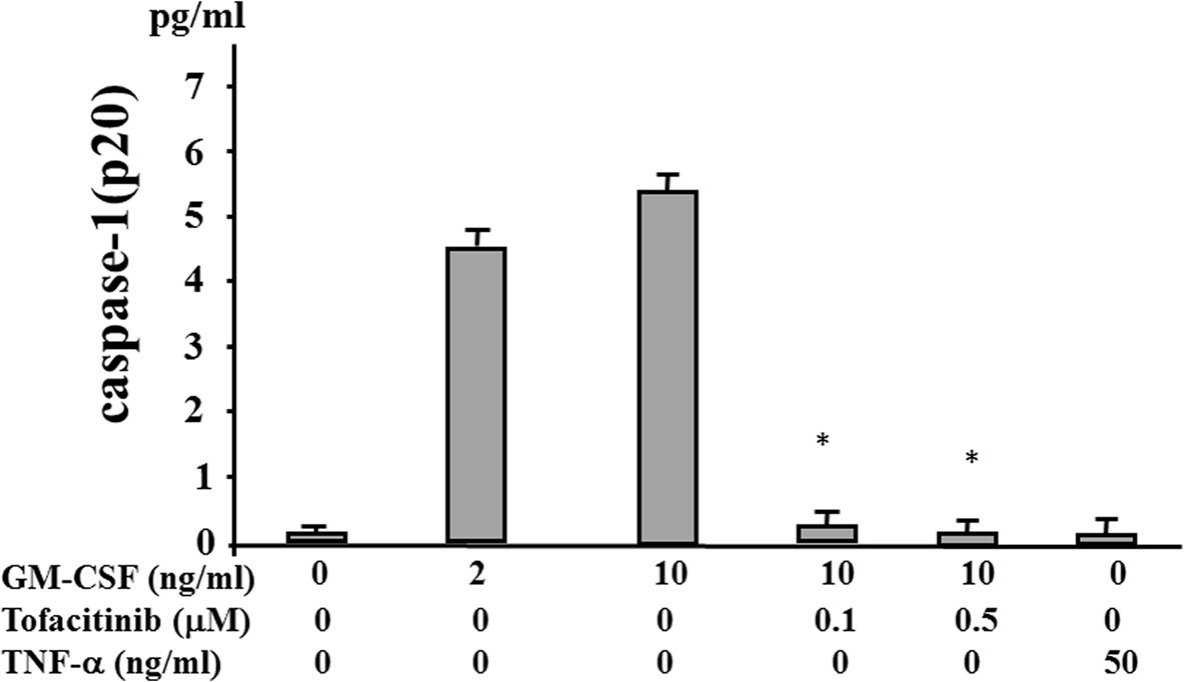
Tofacitinib inhibits the caspase-1 (p20) release from granulocyte–macrophage colony-stimulating factor (GM-CSF)-stimulated neutrophils. Neutrophils (2 × 10^6^/mL) were stimulated with GM-CSF (10 ng/mL) in the presence or absence of the indicated concentrations of tofacitinib for 24 h, and supernatants were analyzed for caspase-1 (p20) by enzyme-linked immunosorbent assay. Values represent the mean ± standard deviation of two independent experiments. *P <0.01 compared with GM-CSF–stimulated neutrophils. Abbreviation: *TNF-α* tumor necrosis factor-alpha

**Fig. 4:**
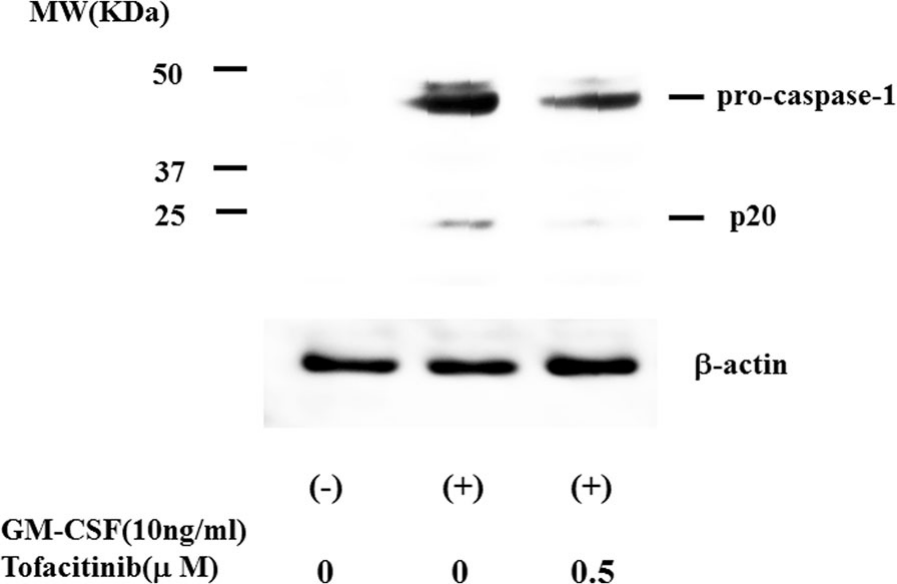
Caspase-1 expressions in granulocyte–macrophage colony-stimulating factor (GM-CSF)-treated neutrophils. Neutrophils were treated with GM-CSF (10 ng/mL) in the presence or absence of tofacitinib for 12 h. Cellular lysates were analyzed by Western blotting by using anti-caspase-1 or anti-β-actin antibodies. Data are representative of two independent experiments. Abbreviation: *MW* molecular weight

We investigated whether cytokine stimulation alone induces IL-1β secretion from human neutrophils. Neutrophils were stimulated with various amounts of TNF-α or GM-CSF, and the supernatants were analyzed for IL-1β by ELISA. The stimulation of human neutrophils with TNF-α alone did not induce IL-1β secretion, but a significant increase in IL-1β secretion occurred following stimulation with GM-CSF (Fig. 1). We also observed that GM-CSF–stimulated IL-1β secretion was completely abrogated by tofacitinib (Fig. 2). During NLRP3 inflammasome activation, a cleaved form of caspase-1 is released along with processed IL-1β [15]. Upon recruitment to an inflammasome complex, caspase-1 is activated and processed into mature caspase-1 formed of p10 and p20 subunits and these subunits have been shown to be secreted and be determined by measuring caspase-1 p20 in culture supernatant [15]. Therefore, we analyzed culture supernatants for the secretion of caspase-1 by using an ELISA specific for the cleaved form of caspase-1 (p20). We found that, consistent with IL-1β production, caspase-1 activation was induced in neutrophils stimulated with GM-CSF but not with TNF-α. Tofacitinib inhibited the GM-CSF–induced increase in caspase-1 secretion from neutrophils (Fig. 3). We examined the expression of caspase-1 by using cellular lysates of GM-CSF–stimulated neutrophils. GM-CSF stimulation upregulated pro-caspase-1 expression as well as cleaved caspase-1 (p20) in neutrophils. Tofacitinib diminished this GM-CSF–induced cleaved form of caspase-1 (Fig. 4). The trypan blue dye exclusion test was performed to check cell viability of GM-CSF–treated neutrophils. However, there was no difference in cell viability assessed by trypan blue exclusion rates between untreated and GM-CSF-treated neutrophils (96.5 ± 4.1% versus 97.5 ± 2–3%, respectively).

### Tofacitinib disrupts GM-CSF signaling in neutrophils

**Fig. 5:**
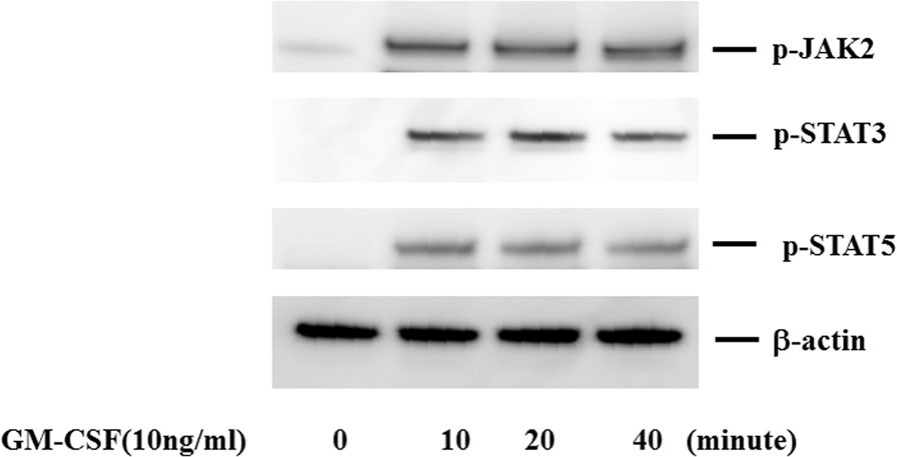
Janus kinase/signal transducer and activation of transcription (JAK/STAT) phosphorylation in granulocyte–macrophage colony-stimulating factor (GM-CSF)-stimulated neutrophils. Neutrophils were stimulated with GM-CSF (10 ng/mL) for the indicated period and lysed. Phosphorylation of JAKs, STAT3, and STAT5 was determined by Western blotting using phospho-specific antibodies against JAK2, STAT1, and STAT3. Phosphorylation of JAK1 and JAK2 was barely detected (data not shown). Three experiments were performed by using rheumatoid arthritis synovial fibroblasts isolated from three different patients with rheumatoid arthritis, and a representative result is shown

**Fig. 6:**
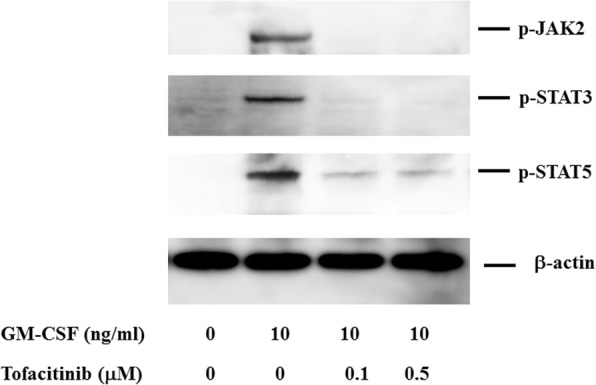
Effects of tofacitinib on Janus kinase/signal transducer and activation of transcription (JAK/STAT) phosphorylation in granulocyte–macrophage colony-stimulating factor (GM-CSF)-stimulated neutrophils. Neutrophils were pretreated with or without tofacitinib for 30 min and then stimulated with GM-CSF (10 ng/mL) for 20 min. Phosphorylation of JAK2, STAT3, and STAT5 was determined by Western blotting using phospho-specific antibodies against JAK2, STAT3, and STAT5. Phosphorylation of JAK1 and JAK3 was barely detected (data not shown)

**Fig. 7:**
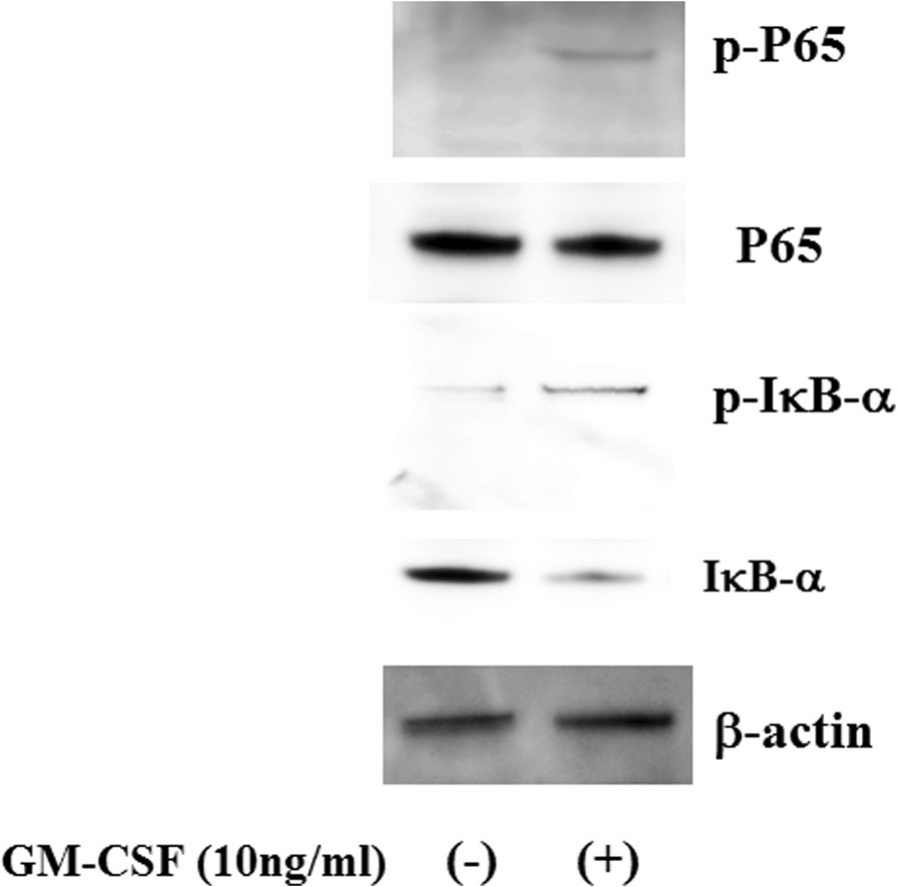
Phosphorylation of nuclear factor-kappa B (NF-κB) p65 in granulocyte–macrophage colony-stimulating factor (GM-CSF)-treated neutrophils. Neutrophils were stimulated with GM-CSF (10 ng/mL) for 20 min. Cells were lysed and cellular lysates were subjected to Western blot using anti-NF-κB, phosphor-NF-κB, IκB-α, phosphor-1κB-α, and β-actin antibodies. Data are representative of two independent experiments

**Fig. 8:**
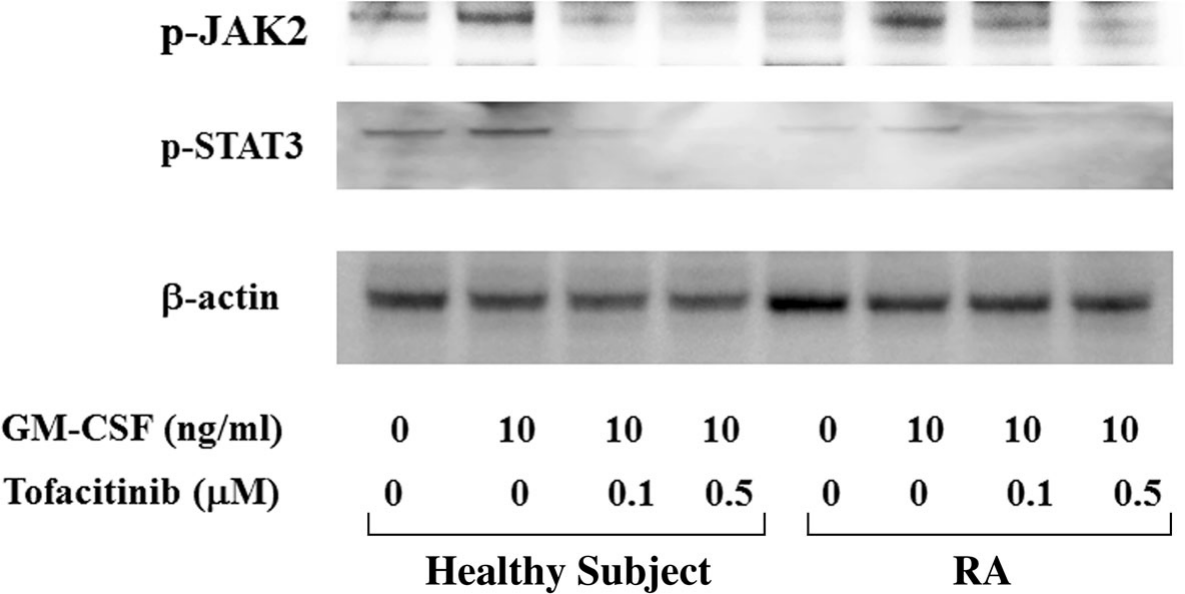
Janus kinase 2/signal transducer and activation of transcription 3 (JAK2/STAT3) phosphorylation in granulocyte–macrophage colony-stimulating factor (GM-CSF)-stimulated neutrophils. Neutrophils from a healthy subject and a patient with rheumatoid arthritis (RA) were pretreated with tofacitinib for 30 min and then stimulated with GM-CSF (10 ng/mL) for 20 min. Cellular lysates were subjected to Western blot using anti-phospho JAK2 or anti-phospho STAT3 antibodies. Data are representative of three independent experiments

**Fig. 9:**
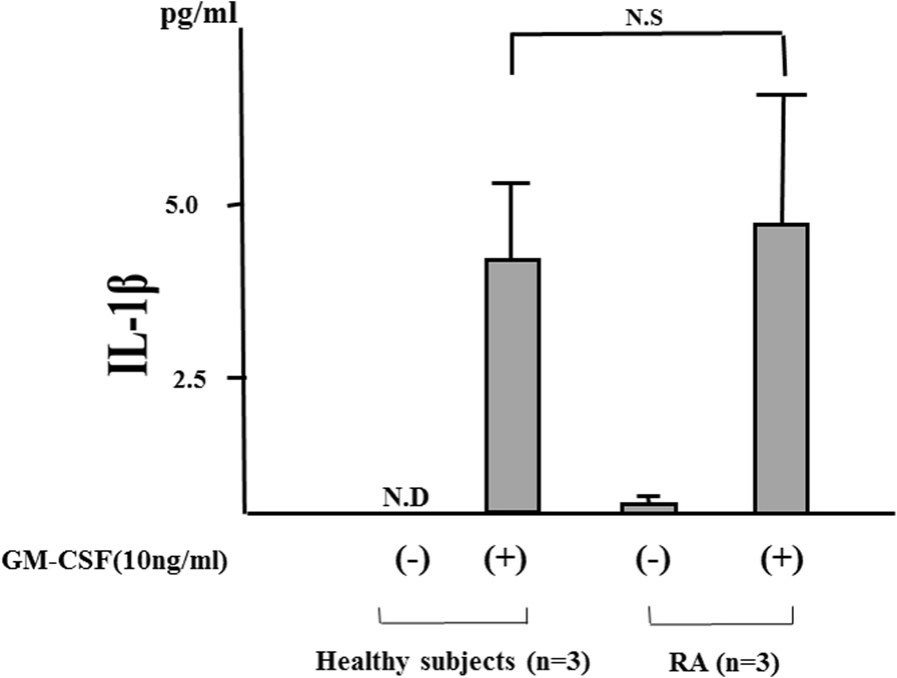
Interleukin-1 beta (IL-1β) secretions from neutrophils isolated from healthy subjects and patients with rheumatoid arthritis (RA). Neutrophils (1 × 10^6^/mL) derived from patients with RA (*n* = 3) or healthy subjects (n = 3) were incubated with or without granulocyte–macrophage colony-stimulating factor (GM-CSF) (10 ng/mL) for 24 h. IL-1β levels in supernatants of neutrophils stimulated with GM-CSF (10 ng/mL) were assessed by enzyme-linked immunosorbent assay. Values were represented as mean ± standard deviation. Abbreviations: *ND* not determined, *NS* not significant

Because tofacitinib abrogates type 1 and type 2 cytokine receptor signaling by blocking JAK kinase, we next investigated whether JAK inhibition interferes with GM-CSF–mediated JAK/STAT signaling. To investigate the effects of tofacitinib on GM-CSF receptor signaling, freshly isolated neutrophils were pretreated with tofacitinib and stimulated with GM-CSF, and protein extracts were analyzed by immunoblotting with phospho-specific antibodies. We examined the phosphorylation status of JAK2 and two important downstream molecules of STAT3 and STAT5. As shown in Fig. 5, JAK2 and STAT3/5 were strongly phosphorylated after 10 min of stimulation with GM-CSF. By contrast, JAK1 or JAK3 phosphorylation was barely detected in GM-CSF–stimulated neutrophils under the same conditions (data not shown), whereas tofacitinib pretreatment (30 min) efficiently inhibited GM-CSF–induced phosphorylation of JAK2 and STAT3/5 (Fig. 6). These results indicate that tofacitinib interferes with the GM-CSF–induced STAT signaling pathway downstream of JAK2. We also examined whether GM-CSF activates the NF-κB pathway in neutrophils. The phosphorylation of NF-κB and 1κB-α was induced in neutrophils by GM-CSF stimulation (Fig. 7). In this result, 1κB-α expression was marginally downregulated and this was probably due to the 1κB-α phosphorylation and subsequent degradation by GM-CSF stimulation. GM-CSF–induced JAK2/STAT3 activation and IL-1β production were compared between neutrophils isolated from healthy subjects and patients with RA. As shown in Fig. 8, GM-CSF stimulation induced JAK2/STAT3 phosphorylation in neutrophils isolated from healthy subjects and patients with RA. Similarly, GM-CSF stimulation induced IL-1β production from neutrophils but its induction did not differ significantly between healthy subjects and patients with RA (Fig. 9).

### GM-CSF induces NLRP3 protein expression in neutrophils

**Fig. 10:**
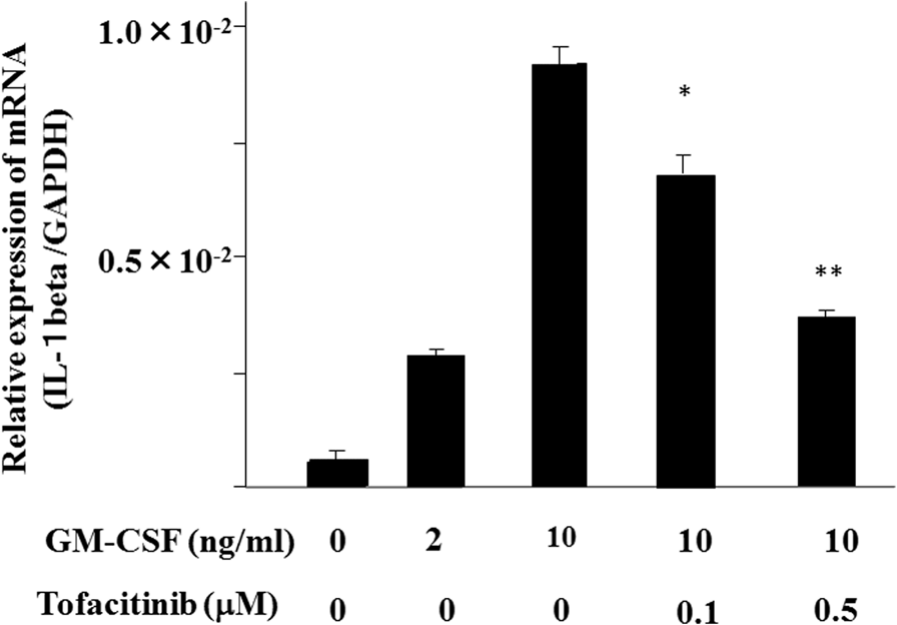
Granulocyte–macrophage colony-stimulating factor (GM-CSF) induces the transcription of pro-interleukin-1 beta (pro-IL-1β) in human neutrophils. Neutrophils were stimulated with GM-CSF (10 ng/mL) in the presence or absence of tofacitinib for 8 h. The cells were harvested and analyzed for pro-IL-1β and glyceraldehydes-3-phosphates dehydrogenase (GAPDH) mRNA levels by real-time polymerase chain reaction. Values represent the mean ± standard deviation of two independent experiments. *P <0.01 compared with GM-CSF–stimulated neutrophils. **P <0.001 compared with GM-CSF–stimulated neutrophils

We next examined whether tofacitinib modulates pro-IL-1β mRNA expression in GM-CSF–stimulated neutrophils. As shown in Fig. 10, GM-CSF is a potent inducer of pro-IL-1β mRNA expression in neutrophils, and tofacitinib could not completely prevent GM-CSF–induced pro-IL-1β mRNA expression Additional file 1. These findings were not consistent with observed IL-1β protein levels, which showed that tofacitinib completely blocked GM-CSF–induced IL-1β secretion from neutrophils.

**Fig. 11:**
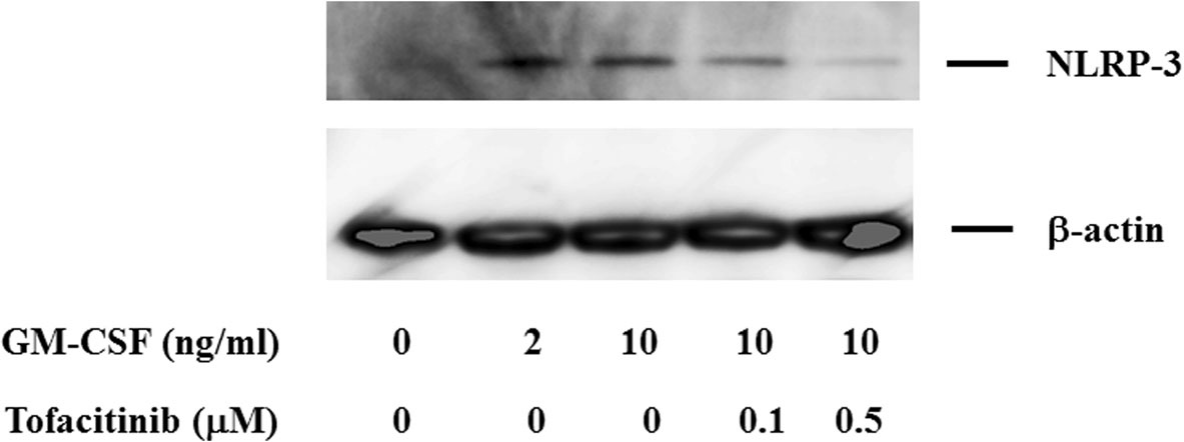
NLR family pyrin domain-containing 3 (NLRP3) expression in neutrophils. Neutrophils were stimulated with granulocyte–macrophage colony-stimulating factor (GM-CSF) in the presence or absence of the indicated concentrations of tofacitinib for 24 h. Cellular lysates were analyzed by Western blot using anti-NLRP3 or anti-β-actin antibodies. Three experiments were performed by using different neutrophils, and a representative result is shown

**Fig. 12:**
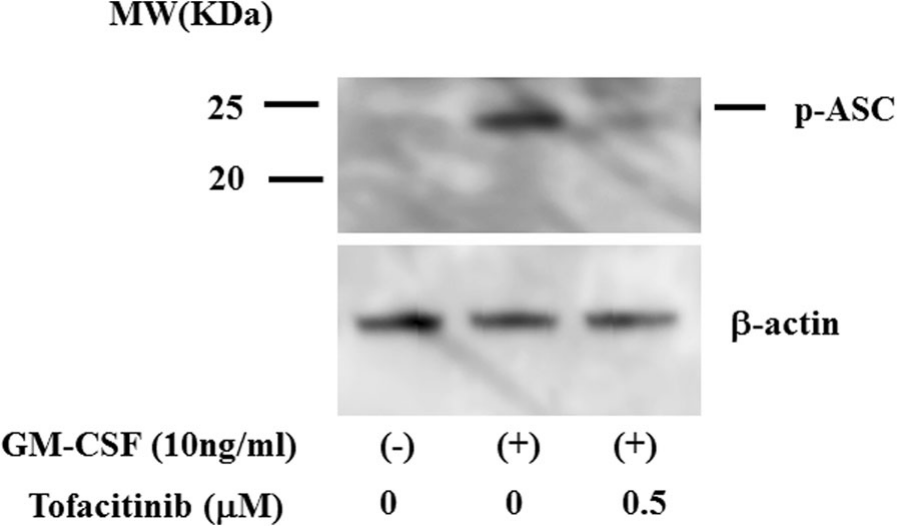
Apoptosis-associated speck-like protein containing CARD (ASC) phosphorylation in granulocyte–macrophage colony-stimulating factor (GM-CSF)-treated neutrophils. Neutrophils were incubated with GM-CSF (10 ng/mL) in the presence or absence of tofacitinib for 8 h. Cellular lysates were subjected to Western blot using anti-phospho-ASC (Tyr-144) or anti-β-actin antibodies. Data are representative of two independent experiments. Abbreviation: *MW* molecular weight

Given the inhibitory effects of tofacitinib on GM-CSF–mediated caspase-1 activation and IL-1β production, we considered the possibility that this JAK inhibitor would also affect NLRP3 inflammasome expression in neutrophils. We therefore investigated the protein expression of NLRP3 in GM-CSF–stimulated neutrophils. Little NLRP3 expression was detected in untreated human neutrophils, whereas GM-CSF stimulation enhanced NLRP3 protein expression and this was inhibited by tofacitinib (Fig. 11). Phosphorylation of Tyr-144 residue of apoptosis-associated speck-like protein containing CARD (ASC) was critical for speck formation and caspase-1 activation during NLRP3 inflammasome activation [16]. We examined whether GM-CSF stimulation induces ASC phosphorylation in neutrophils. The result revealed that ASC could be phosphorylated in response to GM-CSF and tofacitinib diminished this ASC phosphorylation in neutrophils (Fig. 12).

## Discussion

GM-CSF is a well-known hematopoietic factor but its function exceeds that of a simple growth factor [17]. GM-CSF modulates key aspects of both innate and adaptive immunity and plays an important role in the communication between pathogenic auto-reactive T helper (Th) cells and members of the myeloid lineages [18]. In experimental autoimmune encephalitis, the production of GM-CSF by CD4^+^ T cells is required to induce encephalitis [19], while GM-CSF secreted by RA synovial CD4^+^ T cells promotes the differentiation of inflammatory dendritic cells [20]. The success of blocking the GM-CSF receptor in RA therapy suggests that neutralizing the GM-CSF axis could be a useful therapeutic strategy in RA [21].

Activated neutrophils possess many of the molecular properties of macrophages as drivers of inflammatory processes, and several drugs used to treat RA can target neutrophil functions [22]. In this study, we investigated the biological effects of GM-CSF against neutrophils as a major cell of the myeloid lineage. We describe the novel finding that GM-CSF induces inflammasome-dependent IL-1β secretion in human neutrophils. In fact, unlike TNF-α stimulation, GM-CSF stimulation resulted in marked IL-1β secretion and enhanced IL-1β gene expression in neutrophils without the need for a priming signal. Our data strongly suggest that GM-CSF upregulates IL-1β gene expression at the transcriptional as well as the post-translational level in human neutrophils. Furthermore, GM-CSF stimulation was shown to induce the secretion of caspase-1 (p20) in parallel with the upregulation of NLRP3 protein expression in neutrophils. This increased NLRP3 protein expression likely contributes to the heightened IL-1β production following GM-CSF stimulation.

Type 1 and type 2 cytokine receptors lack intrinsic enzymatic activity and associate with a family of cytoplasmic protein tyrosine kinases known as JAKs [23]. Upon cytokine-induced activation, JAKs phosphorylate the cytoplasmic tail of the receptors, leading to the recruitment of STATs, which are also phosphorylated by JAKs [23]. Activated STATs dimerize, translocate to the nucleus, and regulate the expression of target genes [14]. In this study, we demonstrated that tofacitinib, an inhibitor of the JAK family, interferes with JAK2-dependent GM-CSF–driven signaling. It has previously been demonstrated that JAK2 is important in the signal transduction cascade of GM-CSF signaling [13]. Tofacitinib demonstrated selectivity for JAK1 and JAK3 over JAK2 in a whole blood assay in which JAK2 was in its native conformation [24, 25]. Although it is unclear how tofacitinib might spare JAK2 in experiments with isolated JAK kinases, our data clearly showed that tofacitinib efficiently blocked GM-CSF–induced JAK2 phosphorylation in human neutrophils. Tofacitinib was first designed as a JAK3-specific inhibitor, but recent studies suggest that it could be a pan-JAK inhibitor [26].

Our data confirm that neutrophils respond to GM-CSF, which activates JAK2 and the downstream STAT3/STAT5 pathway, resulting in NLRP3 protein expression and IL-1β secretion. Neutrophils are thought to be essential in the pathogenesis of rheumatoid synovitis [27]. Our data suggest that GM-CSF–driven neutrophils play a non-redundant role during the T cell–mediated inflammatory process by polarizing the innate immune activation, including inflammasome activation and IL-1β induction as demonstrated in neutrophils previously [28]. Our data showed that GM-CSF sustains the expression of NLRP3 in neutrophils. Given that NLRP3 expression is known to activate innate immune cells [29], a functional NLRP3 inflammasome resulting in IL-1β induction within the synovium would be expected to contribute to rheumatoid synovitis. Our observations highlight the role of IL-1β by arthrogenic Th cells in the inflamed synovium. Therapeutic interventions targeting GM-CSF during rheumatoid inflammation are likely to restrict not only activated T cells producing GM-CSF but also GM-CSF–responding neutrophils and subsequent IL-1β secretion. This study provides evidence for how GM-CSF impacts on rheumatoid synovitis and which cell type requires a GM-CSF signaling event to mediate inflammation. Pathogenic T cells are the most abundant cellular infiltrates in the rheumatoid synovium and thus cause rheumatoid inflammation [30]. Our data suggest that JAK inhibitors have the potential to block multiple cytokine pathways, including the GM-CSF–mediated autoinflammatory cascade.

There was a limitation in our study. We measured the p20 subunit of caspase-1 in culture supernatants by using caspase-1 p20-specific ELISA. However, Western blot or enzymatic assay should be required to demonstrate the bioactive p20 subunit of caspase-1.

## Conclusion

We have shown that GM-CSF is a strong inducer of IL-1β by activating the inflammasome in neutrophils. Our results indicate that GM-CSF signaling controls the pathogenic expression of IL-1β in neutrophils, which may cause innate cell activation, inflammation, and cartilage damage in RA. Therefore, GM-CSF emerges as a communicator between pathogenic lymphocytes and neutrophils through activating the NLRP3 inflammasome.

## Additional file

Additional file 1: Effects of tofacitinib pretreatment (30 min) on pro-interleukin-1 beta (pro-IL-1β) and NLR family pyrin domain-containing 3 (NLRP3) mRNA expressions in human neutrophils.
Neutrophils were pretreated with or without tofacitinib for 30 min, and cells were harvested and analyzed for pro-IL-1β, NLRP3, and glyceraldehydes-3-phosphates dehydrogenase (GAPDH) mRNA levels by real-time polymerase chain reaction (PCR). Values represent the mean ± standard deviation (SD) of two independent experiments. (TIF 71 kb)

## Abbreviations

ASC: Apoptosis-associated speck-like protein containing CARD
ELISA: Enzyme-linked immunosorbent assay
GM-CSF: Granulocyte–macrophage colony-stimulating factor
IL: Interleukin
JAK: Janus kinase
NF-κB: Nuclear factor-kappa B
NLRP3: NLR family pyrin domain-containing 3
RA: Rheumatoid arthritis
STAT: Signal transduction and activator of transcription
Th: T helper
TNF-α: Tumor necrosis factor-alpha
