# Supplementary figures and images for: Tofacitinib inhibits granulocyte–macrophage colony-stimulating factor-induced NLRP3 inflammasome activation in human neutrophils
Source: Arthritis Res Ther. 2018 Aug 29;20:196. doi: 10.1186/s13075-018-1685-x (PMC6116484; doi:10.1186/s13075-018-1685-x)

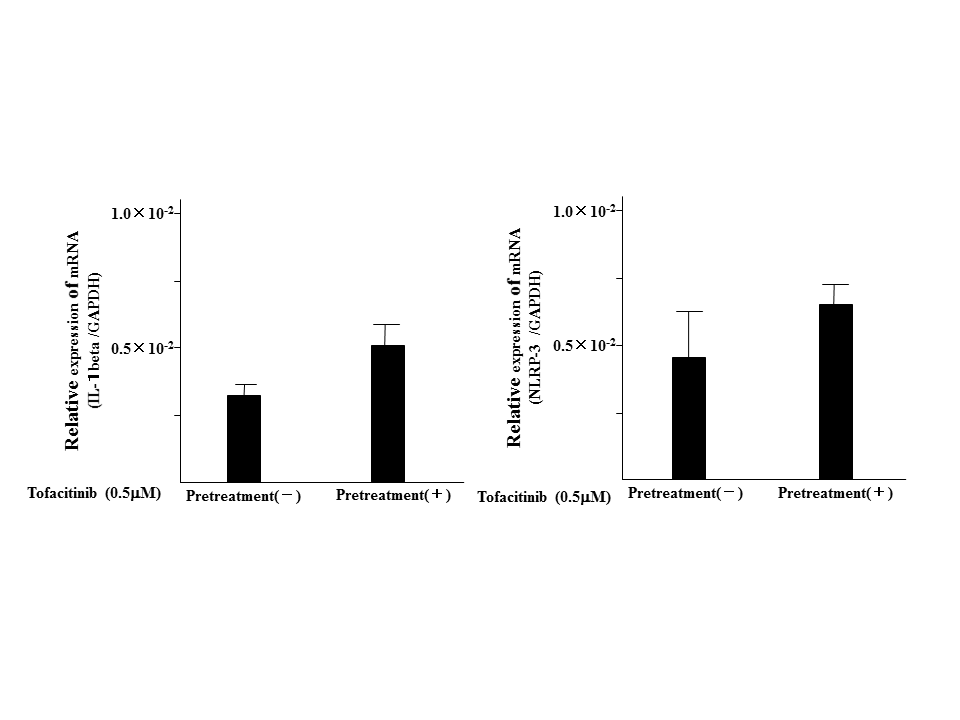

Supplement: Supplementary file 1 — Effects of tofacitinib pretreatment (30 min) on pro-interleukin-1 beta (pro-IL-1β) and NLR family pyrin domain-containing 3 (NLRP3) mRNA expressions in human neutrophils. Neutrophils were pretreated with or without tofacitinib for 30 min, and cells were harvested and analyzed for pro-IL-1β, NLRP3, and glyceraldehydes-3-phosphates dehydrogenase (GAPDH) mRNA levels by real-time polymerase chain reaction (PCR). Values represent the mean ± standard deviation (SD) of two independent experiments. (TIF 71 kb) [file 13075_2018_1685_MOESM1_ESM.tif]
